# A feasibility study of [18F]F-AraG positron emission tomography (PET) for cardiac imaging – myocardial viability in ischemia-reperfusion injury model

**DOI:** 10.21203/rs.3.rs-4244476/v1

**Published:** 2024-04-30

**Authors:** uttam shrestha, Hee-Don Chae, Qizhi Fang, Randall J. Lee, Juliet Packiasamy, Lyna Huynh, Joseph Blecha, Tony L. Huynh, Henry F. VanBrocklin, Jelena Levi, Youngho Seo

**Affiliations:** University of California San Francisco; UCSF: University of California San Francisco; University of California San Francisco; UCSF Medical Center; UCSF: University of California San Francisco; UCSF: University of California San Francisco; UCSF: University of California San Francisco; UCSF: University of California San Francisco; UCSF: University of California San Francisco; UCSF: University of California San Francisco; UCSF: University of California San Francisco

**Keywords:** [18F]FDG, [18F]F-AraG, myocardial infarction, inflammation, cardiac metabolism, T cell infiltration

## Abstract

**Purpose:**

Myocardial infarction (MI) with subsequent inflammation is one of the most common heart conditions leading to progressive tissue damage. A reliable imaging marker to assess tissue viability after MI would help determine the risks and benefits of any intervention. In this study, we investigate whether a new mitochondria-targeted imaging agent, ^18^F-labeled 2’-deoxy-2’-^18^F-fluoro-9-β-d-arabinofuranosylguanine ([^18^F]F-AraG), a positron emission tomography (PET) agent developed for imaging activated T cells, is suitable for cardiac imaging and to test the myocardial viability after MI.

**Procedure:**

To test whether the myocardial [^18^F]-F-AraG signal is coming from cardiomyocytes or immune infiltrates, we compared cardiac signal in wild-type (WT) mice with that of T cell deficient *Rag1* knockout (*Rag1* KO) mice. We assessed the effect of dietary nucleotides on myocardial [^18^F]F-AraG uptake in normal heart by comparing [^18^F]F-AraG signals between mice fed with purified diet and those fed with purified diet supplemented with nucleotides. The myocardial viability was investigated in rodent model by imaging rat with [^18^F]F-AraG and 2-deoxy-2[^18^F]fluoro-D-glucose ([^18^F]FDG) before and after MI. All PET signals were quantified in terms of the percent injected dose per cc (%ID/cc). We also explored [^18^F]FDG signal variability and potential T cell infiltration into fibrotic area in the affected myocardium with H&E analysis.

**Results:**

The difference in %ID/cc for *Rag1* KO and WT mice was not significant (*p* = ns) indicating that the [^18^F]F-AraG signal in the myocardium was primarily coming from cardiomyocytes. No difference in myocardial uptake was observed between [^18^F]F-AraG signals in mice fed with purified diet and with purified diet supplemented with nucleotides (*p* = ns). The [^18^F]FDG signals showed wider variability at different time points. Noticeable [^18^F]F-AraG signals were observed in the affected MI regions. There were T cells in the fibrotic area in the H&E analysis, but they did not constitute the predominant infiltrates.

**Conclusions:**

Our preliminary preclinical data show that [^18^F]F-AraG accumulates in cardiomyocytes indicating that it may be suitable for cardiac imaging and to evaluate the myocardial viability after MI.

## INTRODUCTION

Left ventricular (LV) dysfunction associated with ischemia holds a major clinical significance as this is one of the most common causes of myocardial infarction (MI) and sudden death [1]. Patients after MI are at substantially elevated risks of developing ischemic cardiomyopathy, long-term complications, and comorbidities [2]. Restoration of myocardial perfusion and functional recovery in the aggravated cardiomyocytes is feasible through percutaneous coronary intervention (PCI) such as angioplasty, coronary artery bypass graft (CABG) or other means of therapy. The success rate of these procedures, however, remains controversial due to lack of precise knowledge of tissue viability [3]. Besides, a significant risk is associated with such procedure especially in comorbid patients with multivessel coronary artery disease (CAD) [4]. Nevertheless, there is a notable improvement in patients with documented evidence of myocardial viability after revascularization [5]. It is therefore beneficial to develop a noninvasive imaging marker to determine whether the dysfunctional myocardium is non-viable in which case the risks of intervention would likely be greater than the benefits.

Delineation of a clear boundary between viable myocardium and fibrotic scar in the post-MI patients is clinically challenging [6, 7]. A number of imaging methods have been utilized that enable the localization and semi-quantification of the viability of myocardium [8–11]. The commonly used modalities for assessing myocardial viability are cardiac magnetic resonance (CMR), dobutamine stress echocardiography (ECG), single photon emission computed tomography (SPECT) with ^99m^Tc-Sestamibi, and positron emission tomography (PET) with 2-deoxy-2[^18^F]fluoro-D-glucose ([^18^F]FDG) [12]. [^18^F]FDG PET is routinely used clinically to assess myocardial viability after MI, as it detects tissue with preserved glucose metabolism. However, [^18^F]FDG uptake is affected by blood glucose levels which necessitates careful management of glucose levels before the scan. This task can be particularly challenging in patients with diabetes, a population at higher risk for cardiovascular diseases, including MI.

Since myocardium has immense mitochondrial activity, we hypothesized that a recently developed mitochondria-targeted PET imaging agent, ^18^F-labeled 2’-deoxy-2’-^18^F-fluoro-9-β-d-arabinofuranosylguanine ([^18^F]F-AraG), may be useful in cardiac imaging and assessment of myocardial viability. [^18^F]F-AraG was originally developed for imaging activated T cells [13–15]., and has been evaluated in a number of preclinical models including response to immune checkpoint inhibitor (ICI) therapy [16], acute graft-versus-host disease [17], immunomodulation and tumor profiling [18], arthritis [19] and multiple sclerosis [20]. [^18^F]F-AraG was also investigated in healthy human subjects [21, 22], cancer patients [23] and in COVID–19 convalescent subjects [24].

Here we show, in a preclinical model, that [^18^F]F-AraG accumulates in cardiomyocytes and that it may be suitable to test the myocardial viability after MI. We also explore inflammatory cell infiltration into myocardium post tissue injury, focusing on T cells and assess the effects of dietary nucleotides on [^18^F]F-AraG myocardial signal.

## MATERIALS AND METHODS

All animal procedures were approved by the UCSF Institutional Animal Care and Use Committee (IACUC), and animal housing and care were provided by UCSF Laboratory Animal Resource Center (LARC). All animals were housed in a specific pathogen-free environment and used at the age between 6 to 9 weeks for mice and 4 to 6 months for rats.

### [^18^ F]F-AraG myocardial signal in wildtype (WT) vs. Rag1 knockout (Rag1 KO) mice

To test whether the myocardial [^18^F]F-AraG uptake in normal heart is coming from cardiomyocytes or from T cells, 8 *C57BL/6J* wild-type (WT) mice (6 to 8 weeks old, 4M, 4F) and 4 *C57BL/6J recombination activating gene 1* knockout (*Rag1* KO) mice (6 to 8 weeks old, 4 F) were purchased from Jackson Laboratory (Bar Harbor, ME).

### Effects of dietary nucleotides on [^18^F]F-AraG myocardial signal

We assessed the impact of dietary nucleotides on [^18^F]F-AraG myocardial signal. For this purpose, we obtained 10 *C57BL/6J* female mice (6 to 9 weeks old) from Jackson Laboratory (Bar Harbor, ME), and randomly allocated them into two experimental groups. The mice in the first group were fed a purified diet (AIN-94G purified diet; Envigo, Indianapolis, IN) supplemented with 0.04% (weight/weight) nucleotides (PD + NT) for 4 days, while the mice in the second group were fed a purified diet without nucleotide (PD).

### Rodent model for myocardial infarction

To test whether [^18^F]F-AraG accumulates in viable cardiomyocytes, 6 healthy Sprague-Dawley male rats (4 to 6 months old) were purchased from Charles River Laboratories (Wilmington, MA) and underwent occlusion-reperfusion surgery. Following anaesthetization and intubation, a left-sided thoracotomy was performed to each rat, and the left coronary artery (LCA) was ligated for 120 minutes and reopened to ensure an ischemia-reperfusion induced MI [25]. The LCA ligation prevents mid-distal perfusion causing hypoxia in a moderate to large portion of the distal LV regions resulting in reversible (or irreversible) cardiomyocyte damage. After surgery, the animals were transferred back to animal housing and allowed to recover for about a week until further PET scans were performed.

### [^18^F]F-AraG and [^18^F]FDG myocardial PET imaging

All *in vivo* [^18^F]F-AraG and [^18^F]FDG imaging were performed at different imaging sessions using microPET/CT scanners (Inveon, Siemens Medical Solutions or nanoScan, Mediso USA) with established standard operating procedures. For animal procedure, an angiocatheter was placed into the caudal vein to ensure intravenous administration of radiopharmaceuticals. The catheter placement was checked by flushing a small amount of saline solution.

An approximate dose of 45 MBq/rat [^18^F]F-AraG and 7.5 MBq/mouse of [^18^F]F-AraG were administered intravenously. One hour after [^18^F]F-AraG injection, static PET/CT scans focusing on the heart (15 minutes PET acquisition and 10 minutes CT scan for anatomic reference) were acquired. The same imaging protocol was implemented for [^18^F]FDG imaging with an approximate dose of 30 MBq/rat. Rats were fasted overnight prior to [^18^F]FDG PET scans to reduce plasma glucose levels. All six rats underwent both [^18^F]F-AraG and [^18^F]FDG imaging before surgery for the baseline study. However, only 4 rats were imaged after surgery because of mortality of 2 rats.

PET data were reconstructed using a standard reconstruction algorithm and post-processed with methods provided by the manufacturer. CT-based attenuation correction was also implemented to minimize attenuation artifacts. For image analysis and quantification, data were imported into open source software such as Amide [26]. Whenever quantification is mandated, PET signals were expressed as the percent injected activity per cc (%ID/cc). Volumes of interest (VOIs) were set using the thresholding methods in a semiquantitative fashion. MI volumes were defined with activity below 50% of the peak activity value. For regional assessment, hearts were segmented, and data were analyzed using the 17-segment model of heart (AHA) with the PMOD cardiac PET tool (PCARDP, PMOD technologies, Zurich, Switzerland).

### Immunohistochemistry

Immunohistochemistry and hematoxylin-eosin (H&E) staining were performed by VitroVivo Biotech (Rockville, MD). Frozen sections were fixed with cold acetone/methanol mixture (1:1) for 15 min. Antigen retrieval was performed by heat inactivation in citrate buffer (10 mM citrate buffer (pH 6.0), 0.05% Tween 20; boiled in microwave with high power for 3 min and maintain at 95°C in steamer for 15 min). Following blocking with goat serum, the sections were incubated with rabbit anti-CD3 antibody (#ab16669, 1:800, Abcam, Boston, MA) at 4°C overnight. Then, endogenous peroxidase was blocked with hydrogen peroxide (1% in PBS for 15 min). The sections were then incubated with goat anti-rabbit IgG ImmPRESS^™^ Secondary Antibody for 1 hour at room temperature and subsequently stained using 3,3’ diaminobenzidine and counterstained with Mayer’s hematoxylin solution. Images were captured with a 40x objective on an Olympus VS120 microscope scanner using VS-ASW (Olympus, Japan).

### Statistical Analysis

Any uptake sample quantification was expressed as mean ± SD. Whenever necessary, *p*-values were calculated using two-tailed t-test and Wilcoxon rank-sum test for comparing two independent groups of samples to draw the statistical significance. Any difference was considered statistically significant if the *p*-value was less than 0.05. Any *p*-value less than 0.001 was expressed as *p* < 0.001. All statistical calculations were performed using the Microsoft Excel and open-source statistical package R.

## RESULTS

### [^18^ F]F-AraG myocardial signal in WT vs. Rag1 KO mice

**Figure 1:**
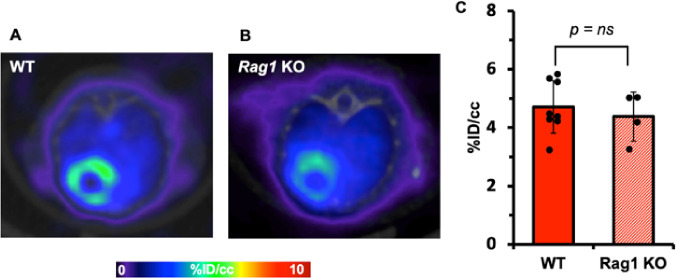
Comparison of [^18^F]F-AraG signals in the myocardium between WT (A) and Rag1 KO (B) mice. The %ID/cc (C) for Rag1 KO tend to be lower compared to WT mice but the difference is not statistically significant (p = ns) indicating myocardial uptake is primarily coming from cardiomyocytes.

As [^18^F]F-AraG was originally developed as a tracer for activated T cells, we first investigated whether the [^18^F]F-AraG signal in the myocardium is coming from cardiomyocytes or T cells. To do so, we compared myocardial [^18^F]F-AraG uptake in wildtype (WT) mice (n = 8; 4F, 4M) with the cardiac uptake in T cell-deficient, *Rag1* KO mice (n = 4; 4F). The %ID/cc for *Rag1* KO was slightly lower compared to WT mice, but the difference was not statistically significant (4.38 ± 0.84 vs. 4.93 ± 0.73, *p* = 0.29) indicating that the [^18^F]F-AraG signal in the myocardium was primarily coming from cardiomyocytes (Fig. 1).

### Comparison of [^18^F]F-AraG and [^18^F]FDG signal variability

**Figure 2:**
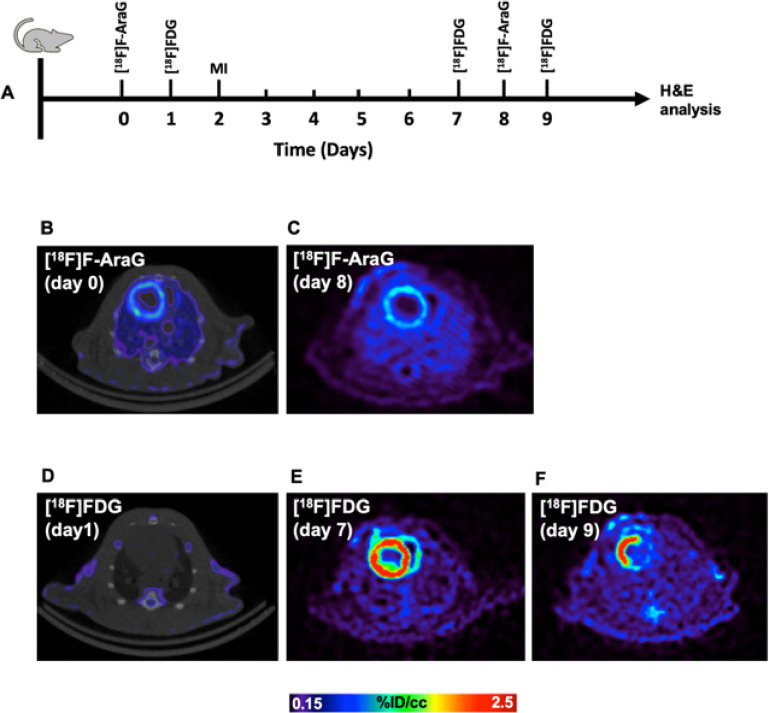
(A) Rat study timeline. Comparison of [^18^F]F-AraG signals in a rat before (B) and after (C) MI. There were no [^18^F]FDG signals in the myocardium before MI (D) while the patterns of uptake differed on two different days after MI (F). All slices were from the midventricular section of the heart.

To better understand utility of [^18^F]F-AraG in cardiac imaging we compared the [^18^F]F-AraG and [^18^F]FDG signals in the myocardium of rat before and after MI. The overall timeline for the rat study and corresponding transaxial slices are shown in Fig. 2. The [^18^F]F-AraG image before MI shows a clear delineation of myocardium with noticeable uptake (Fig. 2C) but the [^18^F]FDG uptake is blunted (Fig. 2D) indicating preferential use of free fatty acids (FFA) as energy substrates in the normal heart [27]. The [^18^F]F-AraG uptake in the myocardium did not vary much after MI (Fig. 2C), while the [^18^F]FDG signals showed wider variabilities at different time points (Figs. 2E, 2F) [28].

**Figure 3:**
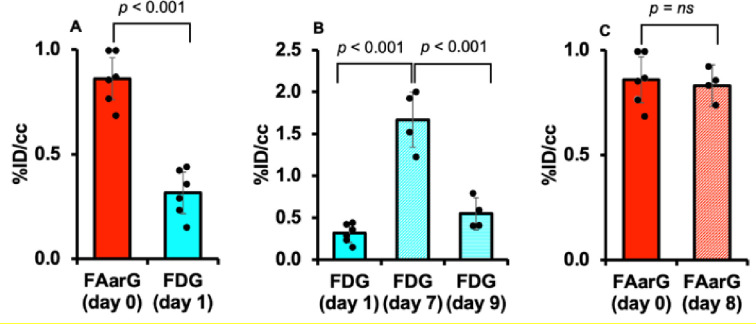
(A) %ID/cc for [^18^F]F-AraG and [^18^F]FDG signals for 6 rats before MI. (B) [^18^F]FDG signal variability in the myocardium at different time points after MI compared to baseline. There were significant differences in [^18^F]FDG signals between different time points. (C) [^18^F]F-AraG signals in the total myocardium tend to decrease after MI from the baseline but the difference was not statistically significant (*p* = ns).

The distribution of [^18^F]F-AraG and [^18^F]FDG myocardial uptakes in terms of %ID/cc are shown in Fig. 3. There was a significant difference in %ID/cc between [^18^F]F-AraG and [^18^F]FDG signals in the normal myocardium (0.86 ± 0.12 vs. 0.32 ± 0.11, *p* < 0.001) (Fig. 3A). The [^18^F]FDG scans preformed before and after MI varied significantly between each time point (Fig. 3B). The variation of [^18^F]FDG signals appears to be related to, in addition to the state and duration of fasting, diet, severity of infarction, the metabolic and hormonal state of each rat at the time of scanning. Although there were reduced activities in the infarct zones, the [^18^F]F-AraG signals in the total myocardium did not show significant variation after MI from the baseline (0.86 ± 0.12 vs. 0.83 ± 0.08, *p* = ns) (Fig. 3C).

### Effects of dietary nucleotides on [^18^F]F-AraG myocardial signal

**Figure 4:**
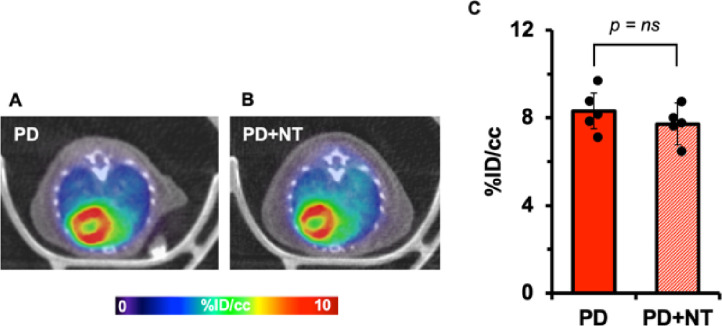
Myocardial [^18^F]F-AraG uptakes in mice fed with purified diet (PD) (A) and diet supplemented with nucleotides (PD+NT) (B). The difference in %ID/cc in two groups was not statistically significant (*p* = ns) indicating that [^18^F]F-AraG uptake in myocardium does not vary with dietary nucleotides (C).

Nucleotides are the building blocks of the nucleic acids and are necessary nutrients to maintain many different cellular functions including the mitochondrial energy metabolism [29]. [^18^F]F-AraG molecules enter cardiomyocytes via nucleoside transporters and get phosphorylated by mitochondrial deoxyguanosine kinase (dGK) resulting in [^18^F]F-AraGTP formation inside inner membrane of mitochondria that are incorporated into mtDNA for biogenesis [16]. To assess whether the myocardial [^18^F]F-AraG uptake in normal heart varies on dietary nucleotides, we compared [^18^F]F-AraG signals between mice fed with purified diet supplemented with nucleotides (PD + NT) with that without nucleotides (PD). No difference in myocardial uptake in terms of %ID/cc was observed between two groups indicating that the nucleotides do not have effect on [^18^F]F-AraG myocardial uptake in normal heart (8.31 ± 0.88 vs. 7.72 ± 0.95, *p* = ns) (Fig. 4).

### Assessing MI with [^18^F]F-AraG and [^18^F]FDG

**Figure 5:**
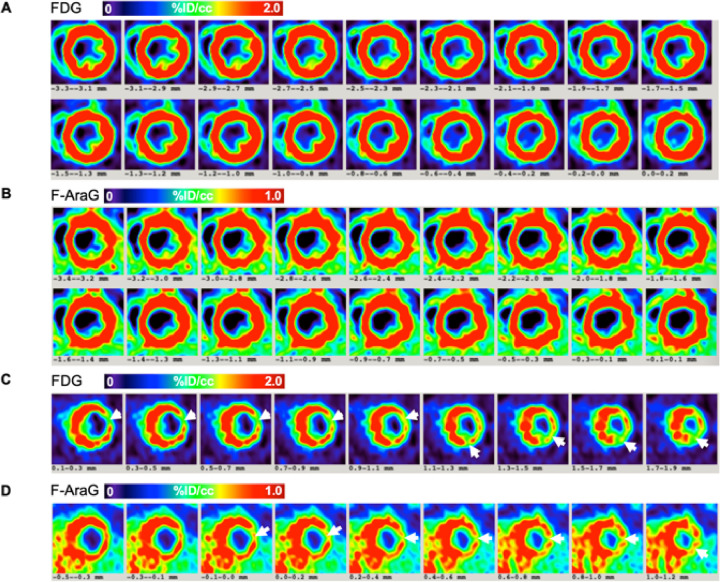
Comparison of [^18^F]FDG (A) and [^18^F]F-AraG (B) signals in the myocardium of a least-affected rat heart after MI. Shown here are the mid ventricular slices in short-axis view. Comparison of [^18^F]FDG (C) and [^18^F]F-AraG (D) signals in the myocardium of another rat with significantly larger MI region. The extent of significantly reduced activity (indicated by arrows) extends from mid-ventricle down to apex in the inferior-lateral wall. Although a significant thinning of myocardial wall was seen in the affected regions by both [^18^F]FDG and [^18^F]F-AraG, the regions of perfusion deficit were smaller in the [^18^F]F-AraG image compared to that of [^18^F]FDG image.

The [^18^F]FDG and [^18^F]F-AraG signals are shown in Figs. 5A and 5B from middle ventricle to apical regions in a minimally affected rat heart following an MI. In this case, the boundary of the MI region was limited to near apex. Figures 5C and 5D display the myocardial slices of another rat with significantly larger MI region. The [^18^F]FDG and [^18^F]F-AraG images shown here are taken from day 7 and 8, respectively. There were significant reduced activities in the inferior-lateral wall in the mid ventricular region down to apex in the [^18^F]FDG signal indicating MI to a broader extent. The arrows in the [^18^F]FDG image slices show the regions with significantly reduced activity affected by the I/R injury that extends up to 5 mm longitudinally. Although the co-registered [^18^F]F-AraG image slices showed a similar pattern of reduced intensity in the affected areas with significant wall thinning, there was a noticeable [^18^F]F-AraG uptake in the MI regions indicating potential mitochondrial activity and thus presence of viable cardiomyocytes.

### T cell infiltration evaluated with IHC H&E staining

**Figure 6:**
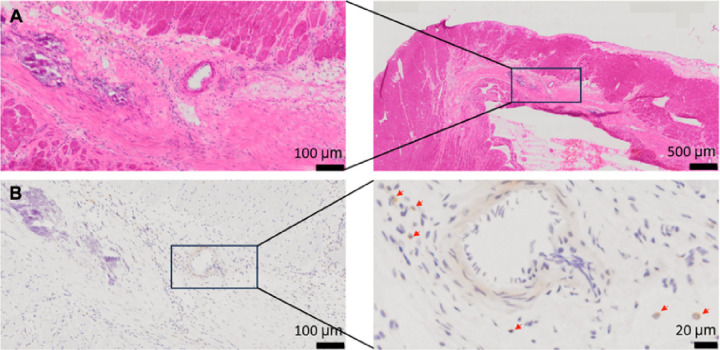
Inflammatory infiltrates in myocardial infarction. The top panels display H&E stain results, highlighting myocardial inflammatory infiltrate and myocardial fibrosis. The bottom panels exhibit immunohistochemistry for CD3, showing the presence of T cells in the infarct area. CD3-positive T cells are specifically indicated with red arrows.

To evaluate immune infiltration in the heart after injury we performed immunohistochemical staining of the hearts extracted one day post imaging. Figure 6 shows an example result from IHC H&E staining analysis for one of the rats. The decreased [^18^F]FDG activity in Fig. 5 in the inferior-lateral wall, from the mid region down to the apex, corresponds to the mid-ventricular section in the H&E staining images, specifically aligning with the area exhibiting fibrosis (Top panels). While immunohistochemistry for CD3 indicates the presence of T cells, indicated with red arrow (Bottom right), in the fibrotic scarred area affected by myocardial infarction, T cells do not constitute the predominant infiltrates (Bottom panels). Out of 4 rats, only 2 showed myocardial fibrosis with the presence of T cells in H&E analysis.

## DISCUSSION

In this study we investigated the interplay between glucose metabolism and mitochondrial biogenesis in the heart after MI. The lower [^18^F]FDG signal in the infarcted region indicates lower glucose metabolism in the injured cardiomyocytes and absence of rampant immune cell infiltration that might be accompanying the injury. The simultaneous reduction of glucose metabolism and mitochondrial activity in the infarct region as demonstrated by matched [^18^F]FDG and [^18^F]F-AraG images may indicate tissue fibrosis and scar buildup [30, 31]. However, presence of [^18^F]F-AraG uptake in the region with substantially reduced [^18^F]FDG uptake may indicate active mitochondrial biogenesis in viable cardiomyocytes.

One of the major functions of mitochondria is to produce energy via oxidative phosphorylation [29]. However, emergent theory suggests that mitochondria not only serve as a cellular power house but also participate in many vital biological processes such as intracellular signaling, pyridine synthesis, phospholipid modifications and calcium regulation [32]. Mitochondrial dysfunction is considered a major orchestrator of cardiomyocyte death after MI. The homeostasis of any healthy cardiomyocyte implies a controlled regulation of mitochondrial activity via enhanced self-renewal (biogenesis) as an adaptive response to external stress such as hypoxia and is vital for the cell survivability [33]. The molecular mechanism behind the role of mitochondria on reducing damage to cardiomyocytes caused by oxidative stress are not fully understood [34]. However, noting the fact that glucose is the ultimate substrate in ischemia because of chronically reduced blood flow, the viable cardiomyocytes that do not take part in the contractility due to loss of metabolism might have persistent mitochondrial biogenesis reflected by the [^18^F]F-AraG activity seen in the infarcted area (Fig. 5).

Ischemic heart disease has been shown to be associated with an excess production of reactive oxygen species (ROS) in the process of oxidation in mitochondria [35, 36]. In this process, the mitochondrial respiratory chain seems to be affected via ROS interference. Normal ROS production is necessary for healthy cellular signaling but its excess may have deleterious effect because it reacts with and damages mtDNA, decreases its copy number, and impairs mitochondrial gene transcription and protein expression via oxidation of large molecules [37]. Toxicity caused by ROS is likely to stimulate the transcription factor and nuclear gene expression required to activate mitochondrial biogenesis by oxidant-driven mechanism [38–40] that [^18^F]F-AraG is deeply associated with.

Mitochondrial biogenesis is also required for activation and proliferation of T cells that may be infiltrating the injured heart. As [^18^F]F-AraG accumulates in activated T cells, signal in the affected area may also be coming from infiltrating lymphocytes as a sign of chronic inflammation [41]. Histological analysis revealed myocardial fibrosis in the infarcted region and presence of T cells (Fig. 6). Considering the relatively low level of T cells infiltrates found in the affected regions, we expect [^18^F]F-AraG accumulation in cardiomyocytes to be the predominant source of signal in the infarcted border zone. Imaging of T cell-deficient *Rag1* KO mice also showed that the [^18^F]F-AraG signal in the normal heart is coming from cardiomyocytes and not from T cells (Fig. 1). Further study is needed to evaluate the [^18^F]F-AraG signal differentiation between cardiomyocytes and T cell after MI.

In healthy subjects, heart gets most of its energy via oxidation of FFA. However, in ischemia, there is an up-regulation of glucose transporters to switch towards glucose metabolism as a main substrate via nonmitochondrial pathway [42]. [^18^F]FDG PET imaging has therefore been routinely used for testing myocardial viability noninvasively [43]. A major limitation of use of [^18^F]FDG PET is its variability on blood glucose level, that depends on diet and duration of fasting, that necessitates a tedious, time-consuming protocol to achieve a diagnostic accuracy [44]. Particularly in diabetic patients, [^18^F]FDG PET has been found to be less efficient due to frequent glucose monitoring and insulin administration. Moreover, the metabolic and hormonal state, which cannot be controlled experimentally, play a role on the [^18^F]FDG variability and may give rise to different uptake values at different time points (Figs. 2, 3). Accumulation of [^18^F]F-AraG in myocardium, by contrast, does not reflect glucose metabolism and may thus represent an [^18^F]FDG PET alternative for viability assessment in diabetic as well as nondiabetic patients. Furthermore, it appears that [^18^F]F-AraG uptake in the myocardium does not differ with the dietary nucleotides, at least in normal myocardium (Fig. 4). However, we do not know if there is variation in [^18^F]F-AraG uptake with the dietary nucleotides in a diseased heart.

Small sample size is a limitation of this study. Like for many other myocardial perfusion agents, [^18^F]F-AraG’s accumulation in the liver affected delineation of the MI region that might have affected the %ID/cc further signifying the importance of respiratory motion correction. Image resolution and partial volume effect are some of the factors that also degraded the image quality which might have affected the [^18^F]FDG – [^18^F]F-AraG image registration. The analysis was performed using predefined thresholds of < 50% cutoff for defining MI region without having auxiliary anatomical imaging such as MR. Other threshold could have resulted in slightly different results and should be included in any future study.

## CONCLUSIONS

The newly developed imaging marker [^18^F]F-AraG holds a great potential for cardiac imaging and assessing myocardial viability after MI. Further study with a larger sample size is needed to verify our preliminary results and to translate the findings to the clinic.

## Data Availability

The data presented in this study are available upon appropriate request to corresponding authors.
